# Supplementary material for: Bacteria hyperpolarize in response to oxygen drop
Source: bioRxiv. 2026 Oct 8:2026.10.07.757406. Preprint. [Version 1] doi: 10.64898/2026.10.07.757406 (PMC13692229; doi:10.64898/2026.10.07.757406)
Supplement: Supplementary file 1 [file NIHPP2026.10.07.757406v1-supplement-6.pdf]

bioRxiv preprint doi: https://doi.org/10.64898/2026.10.07.757406; this version posted October 8, 2026. The copyright holder for this preprint (which was not certified by peer review) is the author/funder, who has granted bioRxiv a license to display the preprint in perpetuity. It is made available under a CC-BY 4.0 International license.

## Tables

Supplementary Table 1

| strain identifier | species | mutation | description |
|---|---|---|---|
| MK0 | *B. subtilis* | | 3610 (WT) |
| JWL6 | *P. megaterium* | | |

bioRxiv preprint doi: https://doi.org/10.64898/2026.10.07.757406; this version posted October 8, 2026. The copyright holder for this preprint (which was not certified by peer review) is the author/funder, who has granted bioRxiv a license to display the preprint in perpetuity. It is made available under a CC-BY 4.0 International license.

| | | | |
|---|---|---|---|
| MK23 | *E. coli* | | MG1655 |
| MK25 | *M. luteus* | | |
| MK32 | *B. subtilis* | Δ*darB* | darB::erm |
| MK33 | *B. subtilis* | Δ*darA* | darA::erm |
| MK34 | *B. subtilis* | Δ*kimA* | kimA::erm |
| MK4 | *B. subtilis* | Δ*cdaA* | cdaA::erm |
| MK8 | *B. subtilis* | Δ*gdpP* | gdpP::erm |
| MK10 | *B. subtilis* | Δ*pgpH* | pgpH::erm |
| MK147 | *B. subtilis* | Δ*fnr* | fnr::erm |
| MK148 | *B. subtilis* | Δ*arfM* | arfM::erm |
| MK149 | *B. subtilis* | Δ*resD* | resD::erm |
| MK150 | *B. subtilis* | Δ*resE* | resE::erm |
| MK151 | *B. subtilis* | Δ*rex* | rex::erm |
| MK152 | *B. subtilis* | Δ*ndh* | ndh::erm |
| MK153 | *B. subtilis* | Δ*yclK* | yclK::erm |
| MK154 | *B. subtilis* | Δ*ldh* | ldh::erm |
| MK155 | *B. subtilis* | Δ*alsR* | alsR::erm |
| MK160 | *B. subtilis* | Δ*yugO* | yugO::erm |
| MK161 | *B. subtilis* | Δ*ktrB* | ktrB::erm |
| MK162 | *B. subtilis* | Δ*ktrD* | ktrD::erm |
| JMJ1217 | *B. subtilis* | Constitutive mScartlet | amyE::Pveg-mScarlet-spec |

Supplementary Table 2

| condition | carbon source | nitrogen source | other |
|---|---|---|---|
| control | 0.5% (v/v) glycerol | 0.125% (w/v) monosodium glutamate | |
| Spec. 2µg/ml | 0.5% (v/v) glycerol | 0.125% (w/v) monosodium glutamate | 2µg/ml spectinomycin |
| 4xGlut. Glyc. | 0.5% (v/v) glycerol | 0.5% (w/v) monosodium glutamate | |
| Glut. Arab. | 68mM arabinose | 0.125% (w/v) monosodium glutamate | |
| Glut. Succ. | 68mM sodium succinate | 0.125% (w/v) monosodium glutamate | |
| Glut. no C | none | 0.125% (w/v) monosodium glutamate | |
| Glut. Fum. | 68mM sodium fumarate | 0.125% (w/v) monosodium glutamate | |
| Glut. Gluc. | 68mM glucose | 0.125% (w/v) monosodium glutamate | |
| Glut. .5xAcet. | 34mM sodium acetate | 0.125% (w/v) monosodium glutamate | |

bioRxiv preprint doi: https://doi.org/10.64898/2026.10.07.757406; this version posted October 8, 2026. The copyright holder for this preprint (which was not certified by peer review) is the author/funder, who has granted bioRxiv a license to display the preprint in perpetuity. It is made available under a CC-BY 4.0 International license.

| | | | |
|---|---|---|---|
| Glut. 1xAcet. | 68mM sodium acetate | 0.125% (w/v) monosodium glutamate | |
| Glutamine Glyc. | 0.5% (v/v) glycerol | 6.7mM L-glutamine | |
| NH4 Succ. | 68mM sodium succinate | 27mM ammonium chloride | |
| NH4 Gluc. | 68mM glucose | 27mM ammonium chloride | |
| NH4Cl Glyc. | 0.5% (v/v) glycerol | 27mM ammonium chloride | |
| NH4 Fum. | 68mM sodium fumarate | 27mM ammonium chloride | |
